# Proximate Composition and Nutritional Value of Three Macroalgae: *Ascophyllum nodosum*, *Fucus vesiculosus* and *Bifurcaria bifurcata*

**DOI:** 10.3390/md15110360

**Published:** 2017-11-15

**Authors:** José M. Lorenzo, Rubén Agregán, Paulo E. S. Munekata, Daniel Franco, Javier Carballo, Selin Şahin, Ramón Lacomba, Francisco J. Barba

**Affiliations:** 1 Centro Tecnológico de la Carne de Galicia, Adva. Galicia n° 4, Parque Tecnológico de Galicia, San Cibrao das Viñas, 32900 Ourense, Spain; rubenagregan@ceteca.net (R.A.); danielfranco@ceteca.net (D.F.); 2 Department of Food Engineering, Faculty of Animal Science and Food Engineering, University of São Paulo, 225 Duque de Caxias Norte Ave, Jardim Elite, Pirassununga, São Paulo 13.635-900, Brazil; pmunekata@gmail.com; 3 Area de Tecnologia de los Alimentos, Facultad de Ciencias de Ourense, Universidad de Vigo, 32004 Ourense, Spain; carbatec@uvigo.es; 4 Department of Chemical Engineering, Engineering Faculty, Istanbul University, Avcilar, 34320 Istanbul, Turkey; selins@istanbul.edu.tr; 5 Grupo Alimentario Citrus (GAC), Avda. dels Gremis, Parcela 28 Pol. Ind. Sector 13 del Túria, Riba-roja de Túria, 46394 València, Spain; ramon.lacomba@uv.es; 6 Nutrition and Food Science Area, Preventive Medicine and Public Health, Food Science, Toxicology and Forensic Medicine Department, Universitat de València, Avda. Vicent Andrés Estellés, s/n, Burjassot, 46100 València, Spain

**Keywords:** *Ascophyllum nodosum*, *Fucus vesiculosus*, *Bifurcaria bifurcate*, seaweeds, fatty acid profile, amino acid content, minerals, chemical composition

## Abstract

Proximate composition (moisture, protein, lipid and ash content) and nutritional value (fatty acid, amino acid and mineral profile) of three macroalgae (*Ascophyllum nodosum*, *Fucus vesiculosus* and *Bifurcaria bifurcate*) were studied. Chemical composition was significantly (*p* < 0.001) different among the three seaweeds. In this regard, the *B. bifurcata* presented the highest fat content (6.54% of dry matter); whereas, *F. vesiculosus* showed the highest protein level (12.99% dry matter). Regarding fatty acid content, the polyunsaturated fatty acids (PUFAs) were the most abundant followed by saturated fatty acids (SFAs) and monounsaturated fatty acids (MUFAs). On the other hand, the three seaweeds are a rich source of K (from 3781.35 to 9316.28 mg/100 g), Mn (from 8.28 to 1.96 mg/100 g), Na (from 1836.82 to 4575.71 mg/100 g) and Ca (from 984.73 to 1160.27 mg/100 g). Finally, the most abundant amino acid was glutamic acid (1874.47–1504.53 mg/100 dry matter), followed by aspartic acid (1677.01–800.84 mg/100 g dry matter) and alanine (985.40–655.73 mg/100 g dry matter).

## 1. Introduction

Seaweeds have been traditionally consumed as food in many cultures, and have been used as condiments, fertilizers, and as source of phycocolloids such as alginate, agar, and carrageenan for industrial applications [1,2,3,4]. Seaweeds are traditionally divided into three main groups corresponding to the phylum: green (Chlorophyta), red (Rhodophyta) and brown (Phaeophyta), depending on their chemical composition and nutritional value [5].

Seaweeds are consumed in Asia as part of the daily diet. Nowadays, brown algae (66.5%) are the most consumed species, followed by red (33%) and green (5%) algae. Today, Japan, China and South Korea, are the countries with the greatest seaweed consumption [6]. Seaweeds are an excellent nutrient source, containing high amounts of macro- and micronutrients [7], as well as bioactive compounds (e.g., catechins such as gallocatechin, epicatechin and catechingallate, flavonols, and flavonol glycosides) with high antioxidant and health beneficial properties [8,9,10]. Food, pharmaceutical, and cosmetic industries have shown interest in the recovery of antioxidant compounds (isolated compounds and/or complex extract mixtures) assisted by conventional (solid-liquid or liquid-liquid extraction, Soxhlet, etc.) and innovative processing technologies (high-pressure, supercritical-CO_2_ (SC-CO_2_), electrotechnologies, microwave- and ultrasound-assisted extraction, among others) [3,9,11]. 

Several authors [12,13,14,15] have reported that the chemical composition of seaweeds varies according to maturity, habitats, environmental conditions, and species. A comprehensive study of nutritional (protein and amino acids, fat and fatty acids, carbohydrates, minerals, and vitamins) and bioactive compounds such as polyphenols, carotenoids, etc., from each seaweed, which can exert some beneficial properties on health, is necessary. There are many brown types of seaweeds found in the Spanish coast and the details of their chemical and nutritional composition are needed in order to fulfil the growing demand for Spanish seaweeds and their derived products. Thus, the aim of the present study was to evaluate the chemical and nutritional properties of three different brown seaweeds *A. nodosum*, *F. vesiculosus* and *B. bifurcata* from the Galician coast.

## 2. Results and Discussion

### 2.1. Chemical Composition of Seaweeds

The proximate composition of the three seaweeds is summarized in Table 1. As can be seen the moisture content showed significant (*p* < 0.001) differences among the three macroalgae, since the lowest value was observed in *B. bifurcata* (7.95%). 

This finding is in close agreement with the data reported by Rodrigues et al. [16], who also noticed that the moisture content of different edible seaweeds species ranged from 8.0 g/100 g of dry weight (DW) in dried *Gracilaria gracilis* to 11.8 g/100 g of DW in dried *Osmundea pinnatifida*. In addition, Gómez-Ordoñez et al. [14] also reported similar moisture contents (between 6.64% and 9.86%) in several edible seaweeds from the northwestern Spanish coast. However, Chan & Matanjun [17] found lower moisture content (5.32%) in freeze-dried *Gracilaria changii* seaweed.

*F. vesiculosus* specie presented the highest protein content (12.99 g/100 DW), followed by *B. bifurcata* (8.92 g/100 DW) and the *A. nodosum* (8.70 g/100 DW). These results are in agreement with the data reported by Fleurence [18], who also noticed low protein content (<15 g/100 DW) in most of the brown seaweeds industrially exploited (*F. vesiculosus*, *A. nodosum*, *Laminaria digitata* and *Himanthalia elongata*). Similar values were found by Gómez-Ordoñez et al. [14] and Alves et al. [19] in *B. bifurcata* (10.92 g/100 DW and 8.57 g/100 g DW, respectively) and by Chan & Matanjun [17] in *G. changii* (12.57 g/100 DW). However, these values were lower than those obtained by Rodrigues et al. [16] for brown (14.4–16.9 g/100 DW), red (20.2–23.8 g/100 DW) and green (18.8 g/100 DW) seaweed species. In addition, our values were lower than those observed by Fleurence [18] in other seaweed species such as *Porphyra tenera* (47 g/100 DW) and *Palmaria palmata* (35 g/100 DW). On the contrary, Sánchez-Machado, López-Cervantes, et al. [20] obtained lower protein content (5.46 g/100 DW) in *H. elongata* dried seaweed. According to Denis et al. [21], the protein content of seaweed changes during the year, having the maximum content during winter and the beginning of spring, and the minimum content during summer and early autumn periods. In addition, the protein level varied among different algal species, geographic areas, seasons, or environmental conditions [22].

In general, seaweeds exhibit low fat content (bellow 4%) [23], which varies significantly through the year [24]. Extractable lipid showed significant (*p* < 0.001) differences among seaweeds, since the highest levels were observed in *B. bifurcata* (6.54% DW). Our values were similar to those reported by Peinado et al. [12], who found the contents ranging from 3.95 to 4.64% DW in *F. vesiculosus* and by Gómez-Ordoñez et al. [14] and Alves et al. [19], who observed fat levels of 5.67% DW and 5.81% DW in *B. bifurcata*, respectively. On the other hand, ash contents were high and ranged from 20.71% DW to 31.68% DW for *F. vesiculosus* and *B. bifurcata*, respectively. These findings are in agreement with the data reported by Alves et al. [19] and Gómez-Ordoñez et al. [14] in *B. bifurcata* (34.31% DW and 30.15% DW, respectively) and by Peinado et al. [12] in *F. vesiculosus* (21–19% DW). The high ash levels constitute an important characteristic of seaweeds, and are higher than those observed in terrestrial vegetables [7]. It is known that high amounts of ash are linked with high levels of minerals.

### 2.2. Mineral Content of Seaweeds

The mineral content of the three macroalgae is given in Table 2. Among the macrominerals, K (3781.35–9316.28 mg/100 g DW) was the most abundant element in the three seaweeds studied, followed by Na (1836.82–4575.71 mg/100 g DW) and Ca (984.73–1160.27 mg/100 g DW). A similar trend was reported by other authors [17,25,26], who found that K was the main mineral element followed by Na. On the other hand, *B. bifurcata* presented a Na/K ratio lower than that observed in the other seaweeds (0.19 vs. 0.58 vs. 1.21, for the *B. bifurcata*, *F. vesiculosus* and *A. nodosum*, respectively), which is really interesting from the nutritional viewpoint, because high Na/K ratio diets and the hypertension incidence are closely linked [27]. Thus, *B. bifurcata* could be useful for the regulation of the Na/K ratio of diets. In addition, Rodrigues et al. [16] suggested that seaweeds with low ratios of Na/K are useful as salt replacers.

The values of manganese in the three macroalgae ranged from 528.04 mg/100 g DW to 867.82 mg/100 g DW, for *B. bifurcata* and *A. nodosum*, respectively, differing significantly (*p* < 0.001) among species. These values were higher than those reported by Chan et al. [17] in *G. changii* (436.13 mg/100 g DW) and lower than the data previously found by Rodrigues et al. [16] in *Sargassum muticum* (1504 mg/100 g DW) and *Codium tomentosum* (1046 mg/100 g DW).

On the other hand, Ca contents also showed significant (*p* < 0.001) differences among seaweeds, showing the highest Ca level in *F. vesiculosus* (1160.27 mg/100 DW). In this regard, Moreiras et al. [28] noticed that Wakame and Sea Spaghetti seaweed species contained approximately eight times more Ca than milk and they could be an excellent source of Ca for the prevention and treatment of osteoporosis, for growing children, and for pre- and post-menopausal women. Phosphorous, the least abundant macromineral, was also detected in *F. vesiculosus* and *B. bifurcata*, ranging from 169.54 mg/100 g DW to 193.57 mg/100 g DW for *F. vesiculosus* and *B. bifurcata*, respectively. 

*A. nodosum* and *F. vesiculosus* also contained iron (ranged from 13.34 mg/100 g DW to 18.99 mg/100 g DW) and Mg (from 1.96 mg/100 DW to 8.28 mg/100 g DW). Our Fe values were higher than those obtained by Rupérez [7] for *Porphyra tenera* (10.3 mg/100 g DW), but less than those found by Rao et al. [29] for *Porphyra vietnamensis* (33 mg/100 g DW). In this regard, *F. vesiculosus* can be a useful to provide the daily intake of iron and to prevent the anemia caused by iron deficiency [30].

### 2.3. Amino Acid Content of Seaweeds

The amino acid (AA) composition of the three seaweeds evaluated is summarized in Table 3. The total AA contents were 7.48, 11.90 and 7.32 g/100 g DW (*p* < 0.001), for *A. nodosum*, *F. vesiculosus*, and *B. bifurcata*, respectively; and these values were comparable to corresponding crude protein levels (Table 1), thus showing that the amount of non-protein nitrogenous materials in these seaweeds was negligible. 

The three seaweeds studied contained all the essential amino acids (EAAs) (excluding cysteine in the *A. nodosum* and *B. bifurcata*). The EAAs content ranged from 3075.28 mg/100 g DW to 5205.23 mg/100 g DW for the *A. nodosum* and *F. vesiculosus*, respectively, showing significant differences among species. The EAA/total AA ratio suggests that more than 40% of the AAs were EAAs. This ratio was lower than the data reported by Chan et al. [17], who observed the ratios (above 55%) in *G. changii*, but comparable to *Porphyra umbilicalis* (36.87%), *Undaria pinnatifida* (42.72%) and *H. elongata* (40.82%) reported by Cofrades et al. [13]. In the essential fraction, leucine was the most abundant, ranging from 524.59 mg/100 g DW to 862.14 mg/100 g DW for *B. bifurcata* and *F. vesiculosus*, respectively, followed by lysine (393.06–800.28 mg/100 g DW), threonine (360.27–613.08 mg/100 g DW) and valine (353.89–582.70 mg/100 g DW). These findings were not in agreement with those reported by Chan et al. [17], who observed that arginine was found to be the highest EAA in *G. changii*, representing 18.69% of the total AAs. On the other hand, glutamic and aspartic acids were the major amino acids found in the non-essential fraction and theses two AAs accounted between 30.67% and 34.20% of the total AAs, for the FV and AN species, respectively. The sum of aspartic and glutamic acids was higher than data reported by other authors [13,17] who found values below 25% in different seaweed species. According to Saini et al. [31], the special flavor and taste of seaweeds in linked to the glutamic and aspartic acids contents. The next highest NEEA were alanine > glycine > serine > proline. Finally, the protein quality of FV seaweed is better than those the other ones, because cysteine is lacking in the AN and BB species.

The nutritional quality of the three seaweeds studied is shown in Table 4. The chemical score (CS) for each of the essential amino acids with respect to the pattern protein, as proposed by Food and Agriculture Organization of the United Nations (FAO)/World Health Organization (WHO)/United Nations (UNU) [32] for humans (children > 1-year old and adults) was calculated.

The profile of the Institute of Medicine, Food and Nutrition (FNB) [33] is also shown for comparative purposes. The analysis of the CS allows the order of the restrictive amino acids to be determined. Concentration of all the essential amino acids were above the FAO/WHO/UNU [32] except for histidine in the *A. nodosum* and *F. vesiculosus* and leucine and lysine in *B. bifurcata*. Thus, histidine was the most limiting AA found in *A. nodosum* and *F. vesiculosus* and lysine seemed to be the limiting AA in *B. bifurcata*. This is in agreement with the data found by Cofrades et al. [13], who found that the most limiting AA in the brown seaweeds was lysine. However, Chan et al. [17] observed that methionine was the most limiting AA found in *G. changii*.

### 2.4. Fatty Acid Profile of Seaweeds

Table 5 shows the fatty acid profile of the three seaweeds studied. The polyunsaturated fatty acids (PUFAs) were the most abundant, ranging from 43.47% to 48.19% for the *A. nodosum* and *F. vesiculosus*, respectively. This result is in agreement with the data previously reported by other authors [13,17,19], who found that PUFAs were the main fatty acids in seaweeds. However, Pen et al. [34] and Maehre et al. [15] observed higher saturated fatty acid (SFA) content in different seaweed species. 

In the present study, the percentage of fatty acid differed significantly (*p* < 0.001) among seaweeds. In this regard, the highest oleic acid (C18:1*n*-9) content (27.83–19.94%) was found in *A. nodosum* and *F. vesiculosus*, whereas *B. bifurcata* presented the highest arachidonic acid (C20:4*n*-6) level (15.24%). A similar trend was reported by Peinado et al. [12] and Ortiz et al. [35], who observed that oleic acid was the main fatty acid in seaweed samples. On the contrary, Chan et al. [17] and Alves et al. [19] reported that docosahexaenoic acid (C22:6*n*-3; DHA) and palmitic acid (C16:0) were the most abundant fatty acids in *G. changgi* and *B. bifurcata*, respectively. These differences on the fatty acid profile could be due to differences among species, as well as other abiotic factors such as light, salinity, and nutrients [36].

Eicosapentaenoic acid (EPA) (C20:5*n*-3) represented from 4.09 to 9.94% of the total fatty acids, whereas docosahexaenoic acid (DHA) was only detected in *B. bifurcata* (11.10% of the total fatty acids). Other studies reported similar EPA percentages in brown algae [12,19,20]. In another work, Maehre et al. [15] found that none of the algae contained DHA, whereas the EPA content varied considerably among species.

On the other hand, Western country diets are deficient in *n*-3 fatty acids due to the low seafood consumption versus the high intake of *n*-6 fatty acid from vegetable oil. In this regard, the World Health Organization (WHO) [30] recommended a *n*-6/*n*-3 ratio below 10. In our study, we observed *n*-6/*n*-3 ratio ranging from 2.62 to 1.41, placing the three macroalgae studied according WHO recommendations. This outcome is in agreement with those reported by other authors [17,19,35] who found *n*-6/*n*-3 ratios between 4.1 and 0.02.

## 3. Material and Methods

### 3.1. Algal Material

The brown seaweeds, *A. nodosum*, *F. vesiculosus* and *B. bifurcata* used in the present study, were kindly supplied by Portomuiños Company (A Coruña, Spain). They were collected from August to September 2015, in the Atlantic Ocean, in the area of Camariñas (A Coruña, Spain). The samples were grinded to obtain powder with a particle size lower than 0.8 mm, using a conventional mincer. Then, the seaweeds were passed through a 0.8 mm mesh sieve and stored under vacuum in plastics bags at −20 °C until analysis.

### 3.2. Chemical Composition

Moisture, protein, and ash were determined following the ISO recommendations (ISO 1442:1997 [37], ISO 937:1978 [38], and ISO 936:1998 [39], respectively). Moisture content was determined by measuring sample (3 g) weight loss at 105 °C in an oven (Memmert UFP 600, Schwabach, Germany), until constant weight. Kjeldahl total nitrogen method was used to determine protein percentage (total nitrogen content was multiplied ×6.25). Five hundred milligrams of seaweed were subjected to reaction with H_2_SO_4_ (CuSO_4_·5H_2_O was employed as a catalyst) in a digester (Gerhardt Kjeldatherm KB, Bonn, Germany), then the organic nitrogen was transformed into (NH_4_)_2_SO_4_, and distilled in alkali condition (Gerhardt Vapodest 50 carroused, Bonn, Germany). Ash content was assessed by determining seaweed (3 g) weight loss in a muffle furnace (Carbolite RWF 1200, Hope Valley, UK) at 600 °C until constant weight. Lipids were determined using the method proposed by Ortiz et al. [35] with some modifications. Lipids from each seaweed (20 g) were extracted with 300 mL of CHCl_3_/CH_3_OH/H_2_O (1:2:0.8), overnight under dark condition. Then, 79 mL of chloroform and 79 mL of water were added to each sample, obtaining a final solvent ratio of CHCl_3_/CH_3_OH/H_2_O of 1:1:0.9 by volume. NaCl (5%) was added and then, samples were centrifuged at 4000 rpm during 10 min. Chloroform phase was concentrated under vacuum condition in order to recover the lipids, which were gravimetrically measured.

### 3.3. Amino Acid Content

Amino acids were extracted following the method proposed by Lorenzo et al. [40]. Amino acids were derived using 6-aminoquinolyl-*N*-hydroxysuccinimidyl carbamate (Waters AccQ-Fluor reagent kit) and determined by RP-HPLC (Waters 2695 Separations Module + Waters 2475 Multi Fluorescence Detector + Waters AccQ-Tag amino acids analysis column). The amino acids content was expressed in mg/100 g of dry matter. 

### 3.4. Protein Quality: Chemical Score of Amino Acids

The chemical score (CS) of the essential amino acids was determined using a protein pattern recommended by FAO/WHO/UNU [32] as reference protein applying the next equation (Equation (1)):

(1) $$CS=\frac{g\;EAA\;in\;tested\;protein}{g\;EAA\;in\;pattern\;protein}\times 100$$

The essential amino acids index (EAA) value was also assessed according to the Equation (2) [40]:

(2) $$EAA=100\times \sqrt[{n}]{\frac{a}{a_{p}}\times \frac{b}{b_{p}}\times \frac{c}{c_{p}}\times \ldots \ldots \frac{j}{j_{p}}}$$

where:

*a*, *b*, *c*, …, *j* = content of Phe, Tyr, Val, Met, Thr, Lys, His, Ile and Leu in seaweeds.
*a_p_*, *b_p_*, *c_p_*, …, *j_p_* = content of Phe, Tyr, Val, Met, Thr, Lys, His, Ile and Leu in protein standard [32].
*n* = number of amino acids used.

### 3.5. Fatty Acid Profile

The lipids extracted (50 mg) were used to determine fatty acid profile. Total fatty acids were transesterified using the method previously by Domínguez et al. [41]. A GC equipment (GC-Agilent 6890 N; Agilent Technologies Spain, S.L., Madrid, Spain) with a flame ionization detector was used for the separation and quantification of the fatty acids methyl esters (FAMEs) using the chromatographic conditions proposed by Domínguez et al. [41]. Individual FAMEs were identified by comparing their retention times with those of authentic standards (Supelco 37 component FAME Mix, Sigma-Aldrich, Barcelona, Spain). C18:1*n*-7 cis (Supelco cis-11-Vaccenic methyl ester), C18:1*n*-11 trans (trans-11-vaccenic methyl ester) and C18:2*n*-7 (CLA) (Matreya LLC Methyl 9(z), 11 (E)-octadecadienoate) were not included in the commercial mix. In addition, nonadecanoic acid (C19:0) was used as internal standard, which was added to the samples prior to methylation. Data were expressed in g/100 g of FAME.

### 3.6. Mineral Profile

The ash samples obtained by ISO recommended standard method [39] were dissolved in 10 mL of 1M HNO_3_. Mineral (Ca, Fe, K, Mg, Mn, Na, P, Zn and Cu) was determined by inductively coupled plasma-optical emission spectroscopy (ICP-OES), using a Thermo-Fisher ICAP 6000 plasma emission spectrometer (Thermo-Fisher, Cambridge, UK), following the method proposed by Lorenzo et al. [42]. All determinations were made in triplicate.

### 3.7. Statistical Analysis

The differences in proximate composition, amino acid, fatty acid and mineral profiles among the three seaweeds studied were examined using an ANOVA test. Least-squares means were compared among seaweeds using the Duncan’s post hoc test (significance level *p* < 0.05). The values were given in terms of mean values ± standard deviations. All statistical analysis were performed using IBM SPSS Statistics^®^ 21 software (IBM Corporation, Armonk, NY, USA).

## 4. Conclusions

Among the three seaweeds studied (*A. nodosum*, *F. vesiculosus*, and *B. bifurcata)*, *B. bifurcata* had the highest level of lipid and ash. It should also be noted that although *B. bifurcata* had the highest total mineral and K contents, *F. vesiculosus* presented the highest Ca, Fe, Mn, and P contents, while *A. nodosum* presented the highest Mg, and Na contents. This fact is of a great importance, especially when seaweeds are used to extract targeted minerals to be used in diets. *F. vesiculosus* had the highest protein content. The three seaweeds studied contained all the essential amino acids (excluding Cys in the *A. nodosum* and *B. bifurcata*). Glu and Asp acids were the predominant amino acids found in the non-essential fraction and theses two amino acids accounted between 30.67% and 34.20% of the total amino acids, for the *F. vesiculosus* and *A. nodosum*, respectively. Concentration of all the essential amino acids were above the chemical score established by FAO/WHO/UNU except for His in the *A. nodosum* and *F. vesiculosus* seaweeds and Leu and Lys in the *B. bifurcata*. Regarding fatty acids, polyunsaturated fatty acid (PUFA) were the predominant fatty acids in the three seaweeds evaluated, ranging from 43.47% to 48.19% for *A. nodosum* and *F. vesiculosus*, respectively. The highest oleic acid content (27.83–19.94%) was found in *A. nodosum* and *F. vesiculosus*, whereas *B. bifurcata* presented the highest arachidonic acid level (15.24%). Moreover, the *n*-6/*n*-3 ratio ranged from 2.62 to 1.41, placing the three macroalgae studied according to WHO recommendations (*n*-6/*n*-3 ratio < 10).

## Figures and Tables

**Table 1:** Proximate composition of the three seaweeds studied (mean ± standard deviation values) (*n* = 5).

Parameters	Seaweed		
	*A. nodosum*	*F. vesiculosus*	*B. bifurcata*
Moisture (g/100 g algae)	11.08 ± 0.53 ^a^	11.23 ± 0.08 ^a^	7.95 ± 0.06 ^b^
Protein (g/100 g DW)	8.70 ± 0.07 ^a^	12.99 ± 0.04 ^b^	8.92 ± 0.09 ^c^
Lipid (g/100 g DW)	3.62 ± 0.17 ^a^	3.75 ± 0.20 ^a^	6.54 ± 0.27 ^b^
Ash (g/100 g DW)	30.89 ± 0.06 ^a^	20.71 ± 0.04 ^b^	31.68 ± 0.41 ^c^

DW: dry weight of seaweed. ^a–c^ Means in the same row not followed by a common superscript letter are significantly different (*p* < 0.05; Duncan test).

**Table 2:** Mineral profile of the three seaweeds studied (mean ± standard deviation values) (*n* = 5).

Minerals (mg/100 g DW)	Seaweed		
	*A. nodosum*	*F. vesiculosus*	*B. bifurcata*
Ca	984.73 ± 47.26 ^a^	1160.27 ± 23.10 ^b^	996.42 ± 12.83 ^a^
Fe	13.34 ± 0.90 ^a^	18.99 ± 0.32 ^b^	n.q.
K	3781.35 ± 13.40 ^a^	3745.05 ± 36.01 ^a^	9316.28 ± 101.94 ^b^
Mg	867.82 ± 12.01 ^a^	732.37 ± 5.35 ^b^	528.04 ± 8.25 ^c^
Mn	1.96 ± 0.69 ^a^	8.28 ± 1.07 ^b^	n.q.
Na	4575.71 ± 50.05 ^a^	2187.51 ± 36.90 ^b^	1836.82 ± 52.12 ^c^
P	n.q.	193.57 ± 1.13 ^a^	169.54 ± 1.41 ^b^
Zn	n.q.	n.q.	n.q.
Cu	n.q.	n.q.	n.q.
Total	10,224.91 ± 64.32 ^a^	8045.96 ± 94.44 ^b^	12,848.97 ± 142.01 ^c^

n.q. = not quantified. DW: dry weight of seaweed. ^a–c^ Means in the same row not followed by a common superscript letter are significantly different (*p* < 0.05; Duncan test).

**Table 3:** Amino acid profile of the three seaweeds studied (mean ± standard deviation values) (*n* = 5).

Amino Acids (mg/100 g DW)	Seaweed		
	*A. nodosum*	*F. vesiculosus*	*B. bifurcata*
**Essential amino acids**			
Threonine	363.22 ± 17.12 ^a^	613.08 ± 33.62 ^b^	360.27 ± 38.25 ^a^
Valine	353.89 ± 32.95 ^a^	582.70 ± 36.73 ^b^	372.82 ± 49.05 ^a^
Methionine	147.59 ± 18.71 ^a^	218.21 ± 20.20 ^b^	178.41 ± 18.08 ^a^
Isoleucine	295.26 ± 25.73 ^a^	507.82 ± 32.42 ^b^	299.73 ± 37.74 ^a^
Leucine	537.37 ± 38.87 ^a^	862.14 ± 57.02 ^b^	524.59 ± 61.38 ^a^
Phenylalanine	340.13 ± 17.74 ^a^	541.53 ± 25.72 ^b^	330.05 ± 32.32 ^a^
Lysine	431.72 ± 38.40 ^a^	800.28 ± 74.20 ^b^	393.06 ± 56.57 ^a^
Histidine	126.46 ± 10.65 ^a^	194.59 ± 8.73 ^b^	138.76 ± 12.70 ^a^
Arginine	316.79 ± 14.05 ^a^	557.87 ± 38.44 ^b^	330.11 ± 42.41 ^a^
Total EAA	2912.42 ± 204.93 ^a^	4878.22 ± 304.12 ^b^	2927.79 ± 346.84 ^a^
**Non-essential amino acids**			
Tyrosine	162.85 ± 24.50 ^a^	327.01 ± 30.59 ^b^	175.00 ± 30.90 ^a^
Asparagine	846.64 ± 38.87 ^a^	1677.01 ± 156.39 ^b^	800.84 ± 105.55 ^a^
Serine	378.62 ± 13.57 ^ab^	630.54 ± 47.00 ^a^	357.10 ± 36.87 ^b^
Glutamic acid	1714.55 ± 133.17 ^a^	1974.47 ± 150.67 ^b^	1504.53 ± 178.74 ^a^
Glycine	417.70 ± 12.89 ^a^	651.24 ± 30.84 ^b^	390.14 ± 29.42 ^a^
Alanine	655.73 ± 34.75 ^a^	985.40 ± 69.50 ^b^	846.65 ± 82.87 ^c^
Proline	399.24 ± 11.70 ^a^	575.19 ± 39.15 ^b^	318.40 ± 40.96 ^c^
Cysteine	0.00 ± 0.00 ^a^	205.23 ± 25.43 ^b^	0.00 ± 0.00 ^a^
Total NEAA	4575.33 ± 198.91 ^a^	7026.10 ± 512.60 ^b^	4392.67 ± 502.38 ^a^
Total AA	7487.76 ± 400.31 ^a^	11,904.32 ± 816.67 ^b^	7320.46 ± 848.14 ^a^
**Relative Amount** EAA (%)	38.87 ± 0.71 ^a^	40.99 ± 0.26 ^b^	39.99 ± 0.31 ^c^

DW: dry weight of seaweed. ^a–c^ Means in the same row not followed by a common superscript letter are significantly different (*p* < 0.05; Duncan test). EAA: Essential Amino acids.

**Table 4:** Nutritional quality of protein for the three seaweeds studied.

Amino Acid	IOM/FNB (2002)	FAO/WHO/UNU (2007)	Seaweeds		
			*A. nodosum* (CS)	*F. vesiculosus* (CS)	*B. bifurcata* (CS)
Histidine	1.8	1.5	96.8	99.8	103.7
Isoleucine	2.5	3.0	113.1	130.3	112.0
Leucine	5.5	5.9	104.7	112.5	99.7
Lysine	5.1	4.5	110.3	136.9	97.9
Met + Cys	2.5	1.6	105.9	203.8	125.0
Phe + Tyr	4.7	3.8	152.1	176.0	149.0
Threonine	2.7	2.3	181.5	125.0	175.6
Valine	3.2	3.9	104.3	115.0	107.2
IAEE			118.4	133.9	118.8

Seaweeds: *A. nodosum* = *Ascophyllum nodosum*; *F. vesiculosus* = *Fucus vesiculosus*; and *B. bifurcate* = *Bifurcaria bifurcata*. Pattern proteins are expressed in (g/100 g protein). Values of CS and IEAA (Index Essential Amino Acids) are referred only respect to FAO/WHO/UNU (2007) protein pattern.

**Table 5:** Fatty acid profile of the three seaweeds studied (mean ± standard deviation values) (*n* = 5).

Fatty Acids	Seaweed		
	*A. nodosum*	*F. vesiculosus*	*B. bifurcata*
C14:0	9.40 ± 0.11 ^a^	11.38 ± 0.11 ^b^	4.52 ± 0.46 ^c^
C14:1*n*-5	0.28 ± 0.00 ^a^	0.10 ± 0.00 ^b^	0.00 ± 0.00 ^c^
C15:0	0.30 ± 0.00 ^a^	0.37 ± 0.00 ^b^	0.17 ± 0.01 ^c^
C16:0	13.42 ± 0.46 ^a^	14.66 ± 0.36 ^b^	17.35 ± 0.43 ^c^
C16:1*n*-7	2.24 ± 0.01 ^a^	1.18 ± 0.02 ^b^	2.51 ± 0.16 ^c^
C17:0	0.41 ± 0.14 ^a^	0.82 ± 0.15 ^b^	0.54 ± 0.02 ^a^
C17:1*n*-7	0.29 ± 0.00 ^a^	0.20 ± 0.00 ^b^	1.87 ± 0.07 ^c^
C18:0	0.76 ± 0.01 ^a^	1.06 ± 0.08 ^b^	1.75 ± 0.13 ^c^
C18:1*n*-11 trans	0.00 ± 0.00 ^a^	0.00 ± 0.00 ^a^	3.57 ± 0.13 ^b^
C18:1*n*-9 cis	27.83 ± 0.26 ^a^	19.94 ± 0.31 ^b^	12.61 ± 0.35 ^c^
C18:1*n*-7 cis	0.45 ± 0.05 ^a^	0.39 ± 0.04 ^a^	0.52 ± 0.03 ^b^
C18:2*n*-6 trans	0.11 ± 0.00 ^a^	0.06 ± 0.00 ^a^	5.68 ± 0.21 ^b^
C18:2*n*-6 cis	7.47 ± 0.12 ^a^	6.43 ± 0.08 ^b^	1.92 ± 0.06 ^c^
C20:0	0.22 ± 0.01 ^a^	0.39 ± 0.01 ^b^	1.89 ± 0.18 ^c^
C18:3*n*-6	0.54 ± 0.01 ^a^	0.56 ± 0.01 ^a^	0.42 ± 0.05 ^b^
C20:1*n*-9	0.07 ± 0.01 ^a^	0.53 ± 0.01 ^b^	4.18 ± 0.12 ^c^
C18:3*n*-3	4.45 ± 0.03 ^a^	7.59 ± 0.11 ^b^	3.97 ± 0.09 ^c^
C18:2*n*-7 (CLA)	0.00 ± 0.00 ^a^	0.00 ± 0.00 ^a^	0.87 ± 0.10 ^b^
C21:0	0.00 ± 0.00 ^a^	0.00 ± 0.00 ^a^	0.71 ± 0.07 ^b^
C20:2*n*-6	5.05 ± 0.02 ^a^	6.46 ± 0.09 ^b^	1.44 ± 0.01 ^c^
C22:0	0.22 ± 0.00 ^a^	0.22 ± 0.00 ^a^	0.34 ± 0.02 ^b^
C20:3*n*-6	0.74 ± 0.04 ^a^	0.69 ± 0.02 ^b^	0.42 ± 0.04 ^c^
C22:1*n*-9	0.00 ± 0.00 ^a^	0.00 ± 0.00 ^a^	0.73 ± 0.04 ^b^
C20:3*n*-3	0.33 ± 0.01 ^a^	0.21 ± 0.00 ^b^	0.00 ± 0.00 ^c^
C20:4*n*-6	17.25 ± 0.26 ^a^	15.86 ± 0.24 ^b^	15.24 ± 0.37 ^c^
C22:2*n*-6	0.29 ± 0.01 ^a^	0.39 ± 0.01 ^b^	1.76 ± 0.09 ^c^
C20:5*n*-3	7.24 ± 0.08 ^a^	9.94 ± 0.14 ^b^	4.09 ± 0.08 ^c^
C24:0	0.41 ± 0.00 ^a^	0.36 ± 0.01 ^b^	0.34 ± 0.03 ^b^
C24:1*n*-9	0.00 ± 0.00 ^a^	0.00 ± 0.00 ^a^	0.53 ± 0.06 ^b^
C22:6*n*-3	0.00 ± 0.00 ^a^	0.00 ± 0.00 ^a^	11.10 ± 1.13 ^b^
SFA	25.14 ± 0.49 ^a^	29.26 ± 0.34 ^b^	27.62 ± 0.77 ^c^
MUFA	31.15 ± 0.23 ^a^	22.33 ± 0.33 ^b^	26.51 ± 0.48 ^c^
PUFA	43.47 ± 0.54 ^a^	48.19 ± 0.62 ^b^	46.91 ± 1.37 ^b^
*n*-3	12.02 ± 0.11 ^a^	17.74 ± 0.25 ^b^	19.16 ± 1.03 ^c^
*n*-6	31.45 ± 0.42 ^a^	30.44 ± 0.38 ^b^	26.87 ± 0.48 ^c^
*n*-6/*n*-3	2.62 ± 0.01 ^a^	1.72 ± 0.01 ^b^	1.41 ± 0.07 ^c^

Results expressed as percentage of total fatty acid analyzed. ^a–c^ means in the same row not followed by a common superscript letter are significantly different (*p* < 0.05; Duncan test). Saturated fatty acids: SFA. Monounsaturated fatty acids: MUFA. Polyunsaturated fatty acids: PUFA.
